# The Role of Natural Killer (NK) Cells in Acute Coronary Syndrome: A Comprehensive Review

**DOI:** 10.3390/biom10111514

**Published:** 2020-11-05

**Authors:** Marko Kumrić, Tina Tičinović Kurir, Josip A. Borovac, Joško Božić

**Affiliations:** 1 Department of Pathophysiology, University of Split School of Medicine, Šoltanska 2, 21000 Split, Croatia; kumricjudo@gmail.com (M.K.); tticinov@mefst.hr (T.T.K.); jborovac@mefst.hr (J.A.B.); 2 Endocrinology Clinic, University Hospital of Split, Spinčićeva 1, 21000 Split, Croatia; 3 Institute of Emergency Medicine of Split-Dalmatia County (ZHM SDZ), Spinčićeva 1, 21000 Split, Croatia

**Keywords:** natural killer cells, inflammation, acute myocardial infarction, cardiac remodeling, coronary artery disease, heart failure

## Abstract

With poor outcomes and an immense financial burden, acute coronary syndrome (ACS) and its ischemic repercussions still present a major global health problem. Unfavorable outcomes seem to be mainly due to adverse cardiac remodeling. Since the inflammatory response takes an important role in remodeling secondary to myocardial infarction (MI), and as inflammation in this manner has not been completely elucidated, we attempted to give rise to a further understanding of ACS pathophysiology. Hence, in this review, we integrated current knowledge of complex communication networks between natural killer (NK) cells and immune and resident heart cells in the context of ACS. Based on available data, the role of NK cells seems to be important in the infarcted myocardium, where it affects heart remodeling. On the other hand, in atherosclerotic plaque, NK cells seem to be mere passers-by, except in the case of chronic infections by atherogenic pathogens. In that case, NK cells seem to support proinflammatory milieu. NK cell research is challenging due to ethical reasons, convergent evolution, and phenotypic diversity among individuals. Therefore, we argue that further research of NK cells in ACS is valuable, given their therapeutic potential in improving postischemic heart remodeling.

## 1. Introduction

Although the introduction of antiplatelet agents and the development of invasive cardiology has previously had a significant impact on reducing mortality and improving outcomes, acute coronary syndrome (ACS) still presents a major public health burden [1]. The financial aspect is even more staggering, as a third of a projected 47 trillion in economic losses to noncommunicable diseases worldwide over the next 20 years will be due to ischemic heart disease (IHD) [2]. Accordingly, further research in this field is mandatory to discover and implement new therapeutic options to reduce expenses and mitigate poor coronary disease outcomes. Poor outcomes are mainly due to adverse left ventricular (LV) remodeling and subsequent heart failure (HF) secondary to myocardial infarction (MI) [3]. LV remodeling in infarct and adjacent noninfarcted regions depends mainly on the inflammatory response. The role of inflammation has not been completely elucidated in atherosclerotic plaque or infarcted myocardium, and further research on this aspect of IHD is necessary [4]. For these reasons, we focused on the role of natural killer (NK) cells, which are poorly discussed and greatly underappreciated in research papers on IHD. 

NK cells are a group of innate immune cells that show spontaneous cytolytic activity against cells under stress, such as virus-infected cells and tumor cells [5]. They belong to the innate lymphoid cells (ILCs) family, a recently discovered group of lymphocytes, and represent about 5–15% of human peripheral blood mononuclear cells (PBMCs) [6]. Except for directly killing target cell through the release of perforin- and granzyme-containing cytotoxic granules, NK cells can also secrete interferon (IFN-γ), tumor necrosis factor (TNF), the granulocyte–macrophage colony-stimulating factor (GM-CSF), and a panel of various immunoregulatory cytokines (IL-5, IL-10, IL-13) and chemokines (CCL-3, CCL-4, CCL-5, CXCL), by which they act as modulators of the inflammatory response [7]. NK cells have recently been recognized for their ability to kill malignant or infected cells and maintain immune homeostasis by killing certain healthy immune cells [6]. Likewise, there is accumulating evidence that NK cells possess memory ability [8,9]. This finding is in contrast to the classical definition of NK cells, by which they belong only in innate immunity cells due to their lack of RAG (Recombination-activating gene) recombinase-dependent clonal antigen receptors [10]. New data suggest that two types of immune memory patterns can be found in NK cells [11]. The first pattern, similarly to B and T cells, is achieved by exerting immunological memory after an encounter with various antigens and the consequent creation of generations of antigen-specific memory NK cells. Secondly, NK cells can remember inflammatory cytokines milieus that imprint long-lasting non-antigen-specific NK cell effector function. These findings of NK cells’ memory could open new horizons in their manipulation and provide us with new therapeutic targets, possibly even in IHD. The main goal of this review was to integrate current knowledge of complex communication networks between NK cells and both immune and resident heart cells in the context of ACS, as well as to address the therapeutic potential of NK cells in the improvement of postischemic heart remodeling for the improvement of outcomes following MI.

## 2. Inflammation in the Acute Coronary Syndrome

### 2.1. In Coronary Arteries

It has been well established that coronary plaque rupture and erosion, with consequent thrombosis and platelet aggregation, are the most important mechanisms by which atherosclerosis leads to acute coronary syndrome [12]. The process begins with changes in the constitutive properties of endothelial cells (ECs) [13,14]. These changes can occur on arterial-susceptible sites due to various triggers: dyslipidemia, microbes, putative antigens, heat shock proteins, low-grade systemic inflammation, and various unrecognized factors [15]. Arterial-susceptible sites are parts of coronary arteries that are more prone to atherosclerotic plaque formation: branch points, the outer wall of bifurcation, and cardiac valves. Observed differences in susceptibility are due to variations in endothelial shear stress (ESS) and/or flow disturbances [16]. In the physiological milieu, vascular ECs maintain a nonthrombogenic interface, have a negatively charged (nonadhesive) surface, and monitor vessel wall permeability [13]. Subtle microenvironmental triggers from blood or interstitia destroy this dynamic equilibrium and activate ECs by modulating their functions. As a result of changes in constitutive properties, dysfunctional ECs begin to synthesize a large array of cytokines and chemokines and upregulate the expression of various cell-adhesion molecules (Table 1). These molecules serve as alarm signals for the recruitment of blood immune cells (monocytes, T-lymphocytes, NK cells, neutrophils and mast cells) and local tissue cells (smooth muscle cells (SMCs) and dendritic cells), thus initiating a robust inflammatory process [17].

Monocytes and T cells adhere to dysfunctional ECs and undergo diapedesis into the intima. In the intimal matrix, monocytes convert to macrophages and lead to the formation of foam cells, whereas lymphocytes switch their synthetic pattern to either proinflammatory (Th1) or anti-inflammatory (Th2 and TREG) [18,19]. It is also worthy to point out the newly recognized role of platelets, which seem to aid leukocytes in transmigration into the intima. Further lesion progression is dictated by the direct or indirect crosstalk between resident and migrated cells within the intima [13], important crosstalk that leads to plaque augmentation between SMCs and immune cells. In response to atherogenic stimuli, SMCs migrate from media to the intima and, along with resident intimal SMCs, change their phenotype from “contractile” to “synthetic.” The latter phenotype produces 25–46 times more collagen than the “contractile” type, expresses a higher number of receptors involved in lipid uptake, and produces various inflammatory mediators, resulting in the creation of a fibrous cap [20,21]. As an overwhelming number of apoptotic cells accumulate inside the plaque, macrophages’ function of clearing them fails (failure of efferocytosis) and, consequently, a lipid-loaded necrotic core is created. The final stage of atherosclerotic plaque formation (and rupture at last) is the unstable fibro-lipid plaque.

The main mechanisms that contribute to rupture are the thinning of the fibrous cap, excess inflammatory cytokines, and proteases, decreased collagen synthesis in SMCs, and the accumulation of cell debris within the necrotic core [13,22]. Macrophages exert their role in plaque rupture by secreting several classes of neutral proteases (matrix metalloproteases (MMPs), serine proteases, cathepsins, urokinase plasminogen activator, tissue plasminogen activator, and plasminogen activator inhibitor-1 and -2). MMPs, alone or in concert with the fibrinolytic system, destroy the matrix either by directly degrading ECM (extracellular matrix) components or indirectly by promoting the death of SMCs and macrophages [23,24,25]. It is important to acknowledge that the role of macrophages in the setting of atherosclerotic plaque is far greater than discussed here [26,27,28]. However, their role is beyond the scope of this review. Mast cells in rupture-prone regions secrete proteases that assist in plaque destabilization, whereas SMCs are involved in plaque rupture due to increased apoptosis and a reduced capacity to synthesize collagen [29,30]. A marked reduction in the ability to synthesize collagen is owed to IFN-γ, a cytokine released either by T-lymphocytes or NK cells [31]. Finally, as rupture occurs, coagulation factors and platelets come in direct contact with exposed ECM rich in proinflammatory factors, such as tissue factor. Consequently, this interaction triggers the coagulation cascade where platelets become activated and aggregate into the thrombus, leading to obstruction of the blood flow to the heart, i.e., acute MI [13].

### 2.2. In Infarcted Myocardium

After acute ischemia of the myocardium occurs, neutrophils enter the ischemic area within 24 h, summoned by an array of inflammatory signals [32]. Apart from their main role in clearing dead cardiomyocytes and their detriments, neutrophils also secrete proteases, release oxidants (via NADPH oxidase), and stimulate a further inflammatory response by secreting various cytokines [33]. Due to the degradation of the extracellular matrix, the myocardium is highly susceptible to rupture in the early postinfarction phase [34]. Following neutrophils, the monocyte lineage enters the necrotic area (Days 2–4). Based on mouse models, it has been established that two subsets of monocytes arrive in the infarcted area sequentially in two phases [33]. The first subset to arrive comprises Ly6C^hi^ monocytes (Phase I), which support inflammation and exert proteolytic and phagocytic functions. After a few days, Ly6C^hi^ are either replaced by or transdifferentiate into the Ly6C^lo^ subset (Phase II). Contrary to Ly6C^hi^, Ly6C^lo^ monocytes have attenuated inflammatory properties and promote myocardial healing leading to myofibroblast accumulation, collagen deposition, and angiogenesis [33]. The mechanisms leading to monocyte accumulation in the heart are different in Phases I and II. During Phase I, the dominance of Ly6C^hi^ monocytes is driven by selective expansion of the Ly6C^hi^ subset. In contrast, dominance of the Ly6C^lo^ subset in Phase II is achieved by the increased preferential recruitment of Ly6C^lo^ monocytes [33]. The balanced expression of these subsets is vital, since they differentially regulate the resolution of inflammation and healing processes, as demonstrated by Panizzi et al. [35]. Although both of these subsets can give rise to macrophages and dendritic cells (Days 7–10), certain monocytes do not differentiate but take part in regulating inflammation. Differentiation of monocytes seems to be influenced by upregulating the macrophage colony-stimulating factor (GM-CSF) [36]. Apart from local signaling, an important regulator of macrophage accumulation and myocardial remodeling is the renin–angiotensin–aldosterone system (RAAS). RAAS activation affects postinfarction healing and heart remodeling on several levels, some of the effects being beneficial, and some deleterious [37]. Except for immediate hemodynamic effects that RAAS exerts after cardiac ischemia occurs, angiotensin II (ATII) plays a role in inflammation by recruiting myelomonocytic cells from the spleen to the ischemic area and steering the crosstalk between monocytes and NK cells [38]. ATII is directly myocytotoxic but can also potentiate myocyte hypertrophy, the growth of vascular smooth muscle cells, and fibroblast proliferation; thus, it has a major impact on cardiac remodeling [39]. It is important to point out that the mentioned effects are achieved by binding to ATII receptor 1 (AT1R), whereas binding to ATII receptor 2 (AT2R) exerts beneficial vascular effects [40]. It has been proposed, but not yet proven, that activation of AT2R during blockage of AT1R may confer additional cardioprotection [41]. Finally, ATII stimulates adrenal glands on the secretion of aldosterone, a potent mineralocorticoid. By binding to mineralocorticoid receptors (MR) on cardiomyocytes, aldosterone acts as a stimulator of the inflammatory response [42]. It has been shown that the immediate application of eplerenone, a selective MR blocker in acute MI complicated by LV dysfunction and HF, improves LV remodeling and significantly reduces adverse events, such as cardiovascular death or hospitalization for cardiovascular events [43]. These effects are achieved by accelerating macrophage infiltration in the infarct zone, improving endothelial vasomotor dysfunction by normalizing nitric oxide (NO)-mediated relaxation, reducing myocardial reactive fibrosis, and transiently upregulating healing-promoting cytokines and factor XIII [44,45]. Fracarollo and colleagues demonstrated that the prevention of macrophage accumulation by liposome-encapsulated clodronate almost abrogated the effects of eplerenone on healing myocardium; therefore, it has been concluded that the benefits of MR blockade are predominantly macrophage-dependent [46].

## 3. The Role of NK Cells in Atherosclerosis

NK cells have been identified within atherosclerotic plaques in humans at an average of 1–2 cells per plaque lesion section [47]. Yet, it is important to acknowledge that understanding the role of NK cells in atherosclerosis is rather challenging because of the lack of representative mouse models of NK cell deficiency. As discussed by Winkels et al., commonly used NK cell deficiency models are all flawed [48]. Beige mutant mice, granzyme A-Ly49A transgenic mice, and (Apoe^−/−^) mice treated with anti-asialo GM serum models, all of which should represent NK cell deficiency, at first lead to the interpretation that NK cells may be proatherogenic. However, beige mice (that carry Lyst gene mutation) seem to have impaired lysosomal storage, which could alter immune cell response. Granzyme A is not exclusive to NK cells as it is expressed by proatherogenic NKT cells and CD8 T cells, whereas asialo GM-1 can also be found on myeloid cells, T cells, and epithelial cells. Using these models, NK cells’ role in atherosclerosis could not be delineated until recently. Nour-Eldine et al. used a precise genetic model of Ncr1^iCre^R26R^lsl-DTA^ mice (NK cells’ deficiency model) and Noe mice (NK cells’ hyper-reactivity model) [47]. Authors determined that neither NK cell deficiency nor hyper-reactivity affects the course of atherosclerosis except under conditions of modeled chronic viral infection (poly(I:C) injections), in which NK cell deficiency was shown to protect against atherosclerosis, implicating the proatherogenic role of NK cells under those terms. The latter could be important for the pathogenesis of coronary artery disease (CAD) in patients suffering from chronic infections. Several human trials have already been conducted on this topic. Hak et al. showed impaired NK cell activity in CAD and a reduction in the CD3-CD56^bright^ cell number [49]. Impairment of NK cell function can lead to increased susceptibility to atherogenic pathogens and promote CAD; however, it can also result in infection by those same pathogens. Increased vulnerability to infections is especially important in elderly people, where NK cells constitute the main mechanism of antiviral defense [50]. Ogata et al. demonstrated that NK cytotoxic activity in the elderly correlates with a history of severe infections or death due to infection [51]. In line with this, an expansion of NKG2C+ NK cells in patients with CAD seems to be associated with the loss of plaque stability in some patients with chronic CMV (Cytomegalovirus) infection [52]. On the other hand, there are several mechanisms by which CAD-associated pathogens interfere with the NK cell compartment. Both CMV and *C. pneumoniae* can lead to the production of IL-10, which quenches cytotoxic NK cell activity by counteracting IL-2 secretion [53,54]. Persistent CMV infection leads to an increase in the CD8+CD28-CD57+ T cell number, a T cell subset which suppresses NK cell function by secreting a non-antigen-specific soluble factor [55]. On the other hand, a reduction in the CD3-CD56^bright^ cell number in peripheral blood, as Hak et al. argue, is due to their migration into the atherosclerotic arterial wall [49]. This is supported by Dalbeth et al., who suggested that CD3-CD56^bright^ cells are capable of migrating to the sites of local inflammation, where they enhance inflammation by stimulating TNF-α production by monocytes [56]. In a recent study, Bonaccorsi et al. observed that atherosclerotic plaques were enriched in CD56^bright^ NK cells compared with autologous peripheral blood [57]. Interestingly, the CD56^bright^ NK cell subset was even more abundant in symptomatic patients, highlighting the possible importance of CD56^bright^ NK cells in plaque instability. In agreement with the latter finding, authors have also observed a higher production of IFN-γ by plaque-resident NK cells. In summary, we argue that the results provided by Bonaccorsi et al., which envisage CD56^bright^ NK cells’ role in the pathophysiology of plaque instability, could only be extrapolated to patients with chronic viral infections. Szymanowski et al. showed that apoptosis of NK cells is increased in patients with CAD, as reflected in the finding that the plasma Fas ligand (FasL), as a measurable marker of cellular apoptosis, significantly correlated with NK cell apoptosis ex vivo in CAD patients. On the other hand, cytokine-induced apoptosis of NK cells resulted in the marked release of FasL, showing that NK cells can be a potential source of soluble FasL. At the same time, FasL seems to regulate the apoptotic susceptibility of NK cells and their levels in CAD [58].

The interaction between activated macrophages and NK cells has been known to trigger an immune response [59]. Cell-to-cell contact and secreted mediators contribute to this crosstalk. When cocultured with macrophages previously exposed to poly I:C, CpG DNA, LPS, or Lacto-N-fucopentaose III, NK cells produce more IFN-γ and upregulate the CD69 marker on their surface [60]. This is achieved through multiple direct cell-to-cell contacts. Firstly, NK cells increase IFN-γ production by LPS-activated macrophages via CD40–CD40L interaction in mice [61]. Although both LPS-activated and inactivated macrophages stimulate NKG2D expression on NK cells, activated macrophages seem to induce NK cells more, highlighting the importance of the NKG2D–NKG2D ligand (RAI-1, MHC class I related chain A (MICA) and UL16-binding protein S) axis in the inflammatory response [60]. Finally, the induction of IFN-γ secretion by NK cells can also be achieved via the interaction between CD2B4 on NK cells and CD48 on LPS-activated macrophages. Based on the exposure dose of LPS, macrophages can elicit different effects on NK cells [60,62]. When exposed to low doses of LPS, macrophages stimulate NK cell proliferation, CD2B4 upregulation, and, finally, their release of IFN-γ. Conversely, when exposed to high doses of LPS, activated macrophages stimulate NK cytotoxicity. In an ex vivo experiment by Dong et al., NK cells produced more IFN-γ when cocultured with dendritic cells previously exposed to oxidized LDL via the CD48–CD2B4 pathway [63]. Unfortunately, except for the latter experiment, these cell-to-cell interactions have not yet been investigated in the setting of vascular inflammation; however, based on the ubiquity of inflammation and available knowledge on atherogenic pathogens, we can hypothesize that it is possible to extrapolate these findings to atherosclerosis. We must be cautious in our predictions, as NK cell activation in humans is in many aspects different from that in mice [64]. Unlike direct cell-to-cell contact, secreted mediators that contribute to the macrophage–NK crosstalk have been, at least to some extent, investigated in vascular dysfunction. Another equally important facet of the crosstalk is the influence of activated NK cells on macrophages, as their mutual relationship creates a positive feedback loop that represents an important augmentation mechanism in the early innate inflammatory response [65]. NK-cell-derived interferon-γ (IFN-γ) stimulated mice to differentiate monocytes to macrophages and inflammatory dendritic cells (DCs). Additionally, IFN-γ caused the replacement of resident mononuclear phagocytes with circulating monocytes that further differentiated into either inflammatory DCs or macrophages. Importantly, these cells further serve as a major source of IL-12, an effect abolished in NK cell depletion [65]. Hence, IFN-γ helps in initiating and augmenting the inflammatory response by driving the local differentiation of monocytes and regulating immune cell dynamics at the inflammation site.

## 4. NK Cells in Acute Coronary Syndrome (ACS)

There is a large discrepancy in scientific data concerning the peripheral blood NK cell number in ACS. Certain authors demonstrated a decline in the peripheral blood NK cell number [62,63,64,65,66,67,68,69], while others demonstrated an elevated number of NK cells in ACS patients compared to healthy controls [70,71]. In studies that compared NK cell levels in ACS and stable angina (SA), most authors found no difference between the two, except for Backtemann et al., who demonstrated a difference at first but failed to repeat it in further studies [66,67]. Backtemann et al. found that in most of the patients, the number of NK cells is restored after one year in both NSTEMI and SA [67]. The recovery in NSTEMI indicates that the NK cell number could reflect disease activity, whereas SA observation could be explained by clinical improvement. In certain patients, the NK cell number failed to reconstitute to that of controls after a one-year follow-up. These patients had a larger waist circumference, triglyceride levels, and remnant cholesterol levels, all indicative of metabolic syndrome. Lynch et al. found that obese patients with metabolic syndrome had lower levels of NK cells compared to obese patients without metabolic syndrome [72]. Increased levels of IL-6 were also observed in patients who failed to restore NK cell levels, which may point towards a lower NK cell proliferation rate [73]. In summary, it seems that the NK cell compartment failed to reconstitute in these patients due to persistent low-grade inflammation, i.e., metabolic syndrome.

Unlike the NK cell number, the data seem to be consistent regarding NK cell function impairment in terms of ACS. Yan et al. measured mRNA expression in both activating and inhibitory receptors on NK cells and found that the expression of both types of receptors was markedly reduced in patients with MI compared to SA and healthy controls [69]. In addition, patients with SA had a lower number of NK cells, similar to those with MI, but with no observed functional impairment. Likewise, several authors demonstrated NK cell function impairment by culturing NK cells extracted from patients with ACS. Reduced NK cell activity against K522 target cells, K562 target cells, and K562 target cells loaded with CFSE was observed, respectively [4,74,75]. It is important to highlight that these observations cannot be completely equated with the NK cytotoxic reactions against autologous cells in vivo. Another important finding by Ortega et al. is the higher percentage of IL-10+ NK cells found in AMI patients. Interestingly, their research showed a negative correlation between IL-10 levels and the severity of the infarction, the TIMI risk score in particular. Additionally, patients with better recovery displayed a reduction in the percentage of IL-10 production. Based on this observation, authors have addressed the importance of IL-10 in regulating NK cell function and the healing of an infarcted heart [4].

NK cells seem to become more activated and susceptible to apoptosis in ACS. Since it has been established that the expression of IL-18R is associated with the upregulated expression of the IFN-γ, the increase of circulating type 1 NK cells (IL-18R+) in ACS compared to SA indicates a more activated state of NK cells in ACS [67,76,77]. NK cells in both non-STEMI and SA patients are more prone to activation by IL-2 compared with controls [74]. Although plasma levels of IL-15 do not differ between patients with ACS and controls, it is hypothesized that IL-15 is upregulated in ACS, indicating the activation of NK cells [67]. The latter is possible, since IL-15 is mostly receptor-bound and plasma levels do not necessarily reflect the true quantity of IL-15 in the organism. Li et al. demonstrated that the number of apoptotic NK cells in blood is higher than in controls, and the NK cells of patients with CAD were more sensitive to oxidized lipids ex vivo, especially 7β-hydroxycholesterol (7betaOH) [78]. Carotenoid levels, which have antioxidant properties and an immunoregulatory role, were generally lower in CAD patients and inversely correlated to spontaneous NK cell apoptosis [79]. Therefore, there are two plausible explanations of NK cell apoptosis, the first being oxidative stress, which failed to be demonstrated in vivo, and the second being increased apoptosis as a consequence of NK cell activation. The mechanism of ineffective degranulation might also explain the functional deficiency of NK cells in the ACS, as demonstrated by Hong et al. [80]. The authors found that NK cytotoxicity was significantly lower in ACS patients compared to healthy controls. This functional deficiency was attributed to an ineffective mechanism of degranulation of toxic granules within NK cells rather than to differences in the expression levels of intracellular cytotoxic granules.

As discussed by Knorr et al., there is an important crosstalk between NK cells and monocytes in ACS [64]. By secreting IL-12 or IL-18, or through direct cell-to-cell contact, macrophages can play a role in NK cell activation, whereas NK cells, by producing IFN-γ, stimulate monocytes on differentiation to macrophages and/or DCs. Macrophages and DCs further produce IL-12 and IL-18 that synergistically stimulate NK cells in the production of IFN-γ, creating a positive feedback loop as aforementioned [81,82]. This crosstalk seems to be steered by the RAAS axis and certain parts of this mutual activation have been disclosed in mice arterial hypertension models [83]. ATII and aldosterone play a regulatory role by stimulating or attenuating the production of chemokines and cytokines from monocytes and/or NK cells. Kossmann et al. demonstrated that IFN-γ^−/−^ and Tbx21^−/−^ mice (mice deficient in the gene encoding for T-bet) were partially protected from ATII-induced vascular endothelial and smooth muscle dysfunction, whereas the depletion of myelomonocytic cell in LysM^iDTR^ mice treated with the diphtheria toxin produced the same effect [84]. Hence, this indicates that ATII-induced vascular damage is both NK-cell- and monocyte-dependent and highlights the importance of the T-box/IFN-γ/IL-12 pathway in this manner.

Furthermore, multiple studies have led to an understanding of the interaction between NK cells and dendritic cells (DC), an interaction that could affect the course of post-MI remodeling. Ayach et al. demonstrated the c-kit-dependent effects of NK cells in cardiac remodeling, which they hypothesize is achieved through the crosstalk between NK cells and DC, leading to a protective paracrine effect [85]. This claim is supported by multiple pieces of evidence. Firstly, c-kit-deficient mice have a reduced NK cell number and cytotoxicity, an effect abrogated by bone marrow transplant (BMT) [85]. Likewise, c-kit-deficient mice have exhibited significantly impaired cardiac function compared to wild-type mice with MI, but this effect was significantly reduced in mice treated with BMT due to the amelioration of adverse heart remodeling. Finally, BMT showed no beneficial effect on the improvement of heart function in c-kit-deficient mice treated with an NK blocker, implying the contribution of NK cells in this process. Additionally, Borg et al. demonstrated that imatinib mesylate (c-kit inhibitor [86]) is able to promote NK cell activation via dendritic cells, but not directly [87].

The beneficial effects of NK cells on cardiac remodeling and heart failure in general were recently discussed by Strassheim et al. [88]. NK cells seem to act protectively against the development of cardiac fibrosis by preventing the accumulation of specific inflammatory populations in the heart and by directly limiting collagen formation in cardiac fibroblasts [89]. By using monocrotaline-induced pulmonary artery hypertension (PAH) rat models, Ormiston et al. and Tamosiuniene et al., respectively, demonstrated that NK cells prevent right ventricular hypertrophy and right ventricular systolic pressure growth, thus indicating their preventive role in this manner [90,91]. According to Edwards et al., NK cell deficiency has been associated with an increased risk of death in PAH patients [92]. Finally, NK cells from PAH patients contribute to vascular remodeling and have higher levels of MMP-9 [89].

## 5. Therapeutic Scope of NK Cells in ACS

NK-cell-derived immunotherapies are already widely investigated as potential targets in malignant patients, since defects in NK cell lineage are well established in a large number of tumors [93]. Several approaches are used in order to induce NK cell response. The transfer of ex vivo activated autologous NK cells has been shown to inhibit tumor progression in renal cell carcinoma (RCC), melanoma, and metastatic breast cancer [94,95,96]. Other approaches include transgenic primary or cell-line-derived NK cells that can express certain receptors and cytokines of choice and the usage of cytokines (IL-2) to stimulate an endogenous NK cell response. Of notice, a large number of NK cells are easily available, either collected from peripheral or cord blood or generated en masse from CD34+ hematopoietic cells from allogeneic or autologous human donors [97]. Additionally, these NK cells can then be genetically manipulated in order to increase the chances of exhibiting only favorable effects. Another therapeutic approach that leads to the enhancement of NK cell function consists of monoclonal antibodies (mAb) directed against immune checkpoint inhibitors such as PD-1, CTLA-4, and Tim-3 [98]. In line with this, NK cell activation can also be achieved by blocking NK inhibitory receptors such as NKG2A/CD94, KIR, or TIGIT. Yet, both of these approaches are so far limited to cancer and liver cirrhosis research [99,100,101]. This pre-existing frame of NK cell-based therapies largely facilitates the implementation of these strategies to ACS treatment.

**Figure 1:**
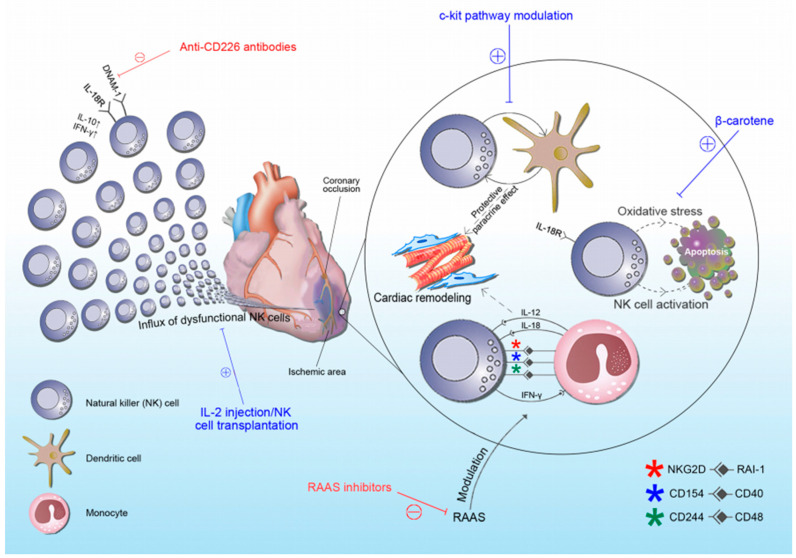
Schematic representation of NK cells’ role in acute coronary syndrome with indicated potential therapeutic targets. Blue lines and “+” represent therapeutic options that stimulate NK cell function, whereas red lines and “−“ represent therapeutic options that inhibit NK cell function. Abbreviations: RAAS: renin–angiotensin–aldosterone system; RAI-1: retinoic-acid-induced 1; DNAM-1: DNAX accessory molecule-1; NKG2D: natural killer group 2D, CD: cluster of differentiation; IFN-γ: interferon-γ.

So far, few studies have tested the therapeutic potential of NK cells in myocardial infarction. Bouchentouf et al. used recombinant human interleukin (rhIL-2) injections two days following myocardial infarction in immunocompetent (C57Bl6) and immunodeficient (NOD-SCID IL2Rγnull) mice [102]. They demonstrated that in immunocompetent mice, rhIL-2 injection resulted in the preservation of LV fractional shortening and remodeling, reduced cardiac fibrosis, and enhanced angiogenesis in the infarcted and border zones of the myocardium. In immunodeficient mice, these effects were not observed. As the proangiogenic effect of rhIL-2 correlated with the infiltration of NK cells, the authors concluded that IL-2 benefits are, at least partially, NK-cell-dependent. In a recent study, Li et al. observed that CD226 expression is dramatically increased in the infarcted heart area and CD226 deletion improves postinfarction healing and cardiac function in mice [78]. CD226, also known as DNAX accessory molecule 1 (DNAM-1), is a membrane protein that belongs to the immunoglobulin superfamily of receptors and is constitutively expressed on the majority of T cells, NK cells, and monocytes [103]. The role of DNAM-1 as an activating receptor and in NK cell education has been well studied, yet its role in ACS is poorly understood [104,105,106]. Authors have concluded that the beneficial effects of CD226 depletion are achieved by favoring macrophage polarization towards a reparative phenotype. We hypothesize (based on our unpublished observations) that CD226-expressing NK cells are, at least to some extent, involved in the process. Another possible approach to NK cell function enhancement and consequent myocardial healing improvement comprises beta carotenes. As previously mentioned, patients with CAD exhibit lower levels of carotenoids [79]. Lidebjer et al. showed that levels of oxygenated carotenoids are associated with the proportions of NK cells in blood [107]. Finally, in a study by Santos et al., beta-carotene supplementation resulted in enhanced NK cell activity in elderly males [108]. It is so far speculative, but it seems that optimal levels of carotenoids could ameliorate post-MI outcomes by restoring NK cell function. As new data emerge, more and more therapeutic options, including NK cell manipulation, arise. Given its role in myocardial healing, IL-10 has recently shown potential benefits, but further research is needed to determine if this could be a new therapeutic target. Perhaps the most important setback in implementing NK-cell-based therapies is timing. NK cells by nature have a dual role in the healing process, as they act actively cytotoxic in one phase but differentiate to regulatory cells that produce cytokines supportive of tissue repair in the other phase [109]. The true mastership would be to apply NK-cell-based therapy to the point at which NK cells will exert their reparative function. This has been very challenging so far, as we still do not possess proper diagnostic tools. Hence, we are unaware of the inflammatory/reparative phase in which the healing myocardium really is. Another potential problem of NK-cell-based therapy is the diversity of NK cells [64]. Most studies use splenic and/or peripheral blood NK cells, whereas tissue-resident NK cells, which often have a completely different cytokine and receptor repertoire, are mostly neglected [109]. We believe that strategies that use the blockage of inhibitory pathways to increase NK cell function could, at least partially, solve this problem, as they are more likely to stimulate tissue-resident NK cells. In Figure 1, we summarized potential NK-cell-based therapeutic targets in postischemic cardiac remodeling (Figure 1).

## 6. Conclusions

In this review, we attempted to unravel the complex interaction of immune cells in both coronary plaques and infarcted myocardium and thereby address the importance of NK cells in the pathophysiology of ACS, with the intention to support investigations of their therapeutic potential. Based on available data, the role of NK cells seems to be important in the infarcted myocardium, where it affects heart remodeling. In atherosclerotic plaque, NK cells seem to be innocent bystanders, except in the case of chronic infections caused by atherogenic pathogens. Unfortunately, further research on NK cells has several major setbacks. The possible role of local NK cells in ischemic myocardial injury during one’s lifetime cannot be sufficiently investigated due to ethical factors in the tissue collection. Furthermore, due to their convergent evolution, mouse NK cell subsets do not completely reflect those of humans, making it more difficult to draw conclusions. Finally, NK cells display extensive phenotypic diversity between individuals, which highlights the difficulty in assessing the NK cell phenotype in any particular pathology. However, owing to the massive therapeutic potential it possesses, we support further research on NK cells in terms of ACS.

## Figures and Tables

**Table 1:** Expression of molecules in the course of atherosclerotic plaque development.

Cell Type		Associated Molecules
**Monocyte**	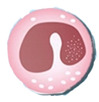	TNF-α, tissue factor, tPA, uPA, PAI-1,2, CSF1, MIF, MCP-1, IL-1,6,15,20, PDGF, VEGF, resistin, NO, TIMP-1,2,3
Dysfunctional EC	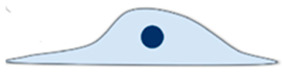	P-selectin, CD106, CD54, TNF-α, IL-1, 6,8,11,14,15,18, fibronectin, collagen type IV, von Willebrand factor, PAI-1, PAF, thrombomodulin, tPA, urokinase, TFPI, MCP-1, CCL5, MIF, CSF1, CSF2, NO, endothelin, MMP-1, 2, TIMP-2, HMGB1, HSP 60, laminin, versican, perlecan
NK cell	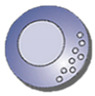	IFN-γ, NKG2C, NKG2D, TRAIL, LILRB1, CD244, IL-18
Dendritic cell	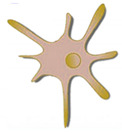	TNF-α, CCL19, IFN-α, IL-6,12, MMP-9

Abbreviations: EC: endothelial cell; TFPI: tissue factor pathway inhibitor; MIF: macrophage migration inhibitory factor; LILRB1: leukocyte immunoglobulin-like receptor subfamily B member 1; PAF: platelet-activating factor; TIMP-1,2,3: tissue inhibitor of metalloproteinases 1, 2, 3; MCP-1: monocyte chemoattractant protein-1; MMP-1,2: matrix metalloproteinase 1, 2; TRAIL: TNF-related apoptosis-inducing ligand; CD: cluster of differentiation; HMGB1: high-mobility group box 1; HSP 60: heat shock protein 60; tPA: tissue plasminogen activator; PDGF: platelet-derived growth factor; VEGF: vascular endothelial growth factor; NO: nitric oxide; CSF1: colony-stimulating factor 1; CSF2: colony-stimulating factor 2; NKG2C, 2D: natural killer group 2C, 2D.
